# Research progress of nanoparticles in diagnosis and treatment of hepatocellular carcinoma

**DOI:** 10.1515/biol-2022-0932

**Published:** 2024-08-23

**Authors:** Lijun Xing, Yun Chen, Tingting Zheng

**Affiliations:** Shenzhen Key Laboratory for Drug Addiction and Medication Safety, Department of Hubei University of Medicine, Institute of Ultrasonic Medicine, Peking University Shenzhen Hospital, Shenzhen Peking University-Hong Kong University of Science and Technology Medical Center, Shenzhen, 518036, P. R. China; Shenzhen Key Laboratory for Drug Addiction and Medication Safety, Department of Ultrasound, Institute of Ultrasonic Medicine, Peking University Shenzhen Hospital, Shenzhen Peking University-Hong Kong University of Science and Technology Medical Center, Shenzhen, 518036, P. R. China

**Keywords:** hepatocellular carcinoma, nanoparticles, precision therapy, integrated treatment

## Abstract

Hepatocellular carcinoma (HCC) is among the most common malignant liver tumors. Despite progress in anticancer drugs and surgical approaches, early detection of HCC remains challenging, often leading to late-stage diagnosis where rapid disease progression precludes surgical intervention, leaving chemotherapy as the only option. However, the systemic toxicity, low bioavailability, and significant adverse effects of chemotherapy drugs often lead to resistance, rendering treatments ineffective for many patients. This article outlines how nanoparticles, following functional modification, offer high sensitivity, reduced drug toxicity, and extended duration of action, enabling precise targeting of drugs to HCC tissues. Combined with other therapeutic modalities and imaging techniques, this significantly enhances the diagnosis, treatment, and long-term prognosis of HCC. The advent of nanomedicine provides new methodologies and strategies for the precise diagnosis and integrated treatment of HCC.

## 1 Introduction

Liver cancer ranks among the most prevalent malignancies worldwide, with global new cases surpassing 900,000 annually as of 2020 [1]. Projections suggest this number may reach one million by 2025. Hepatocellular carcinoma (HCC) constitutes approximately 90% of liver cancer cases, with incidence rates in developing countries two to three times higher than in developed nations [2,3]. Risk factors for HCC include liver cirrhosis induced by fibrosis and inflammation, aflatoxin-induced toxicity, smoking, metabolic disorders including hereditary hemochromatosis, diabetes, non-alcoholic fatty liver disease, and immune-related diseases such as primary biliary cirrhosis and autoimmune hepatitis [4,5]. Currently, clinical treatment options for HCC include surgical resection, ablation, liver transplantation, radiotherapy, transarterial chemoembolization (TACE), combined therapy, and chemotherapy, with the choice of treatment dependent on the clinical staging and specific diagnosis of HCC. Early-stage patients benefit most from surgical resection and liver transplantation. As the disease progresses, radiation or combined therapy serves as a frontline treatment for HCC, but resistance development limits their efficacy, and chemotherapy introduces various adverse effects [6,7]. For late-stage HCC, systemic treatments such as TACE and oral sorafenib are popular, improving the 2-year survival rate by 23%. However, less than one-third of patients benefit from these treatments, with resistance evident within 6 months of initiating therapy [8]. Given the typical late diagnosis of HCC, the prognosis remains poor, with only 25–30% of cases diagnosed at an early stage and a mere 5–15% suitable for surgical resection, highlighting the importance of early diagnosis and treatment [3,9]. Current clinical detection methods, including imaging and tumor markers, struggle to identify early-stage HCC, especially lesions ≤1 cm, which lack clinical signs and exhibit atypical pathology.

**Figure 1:**
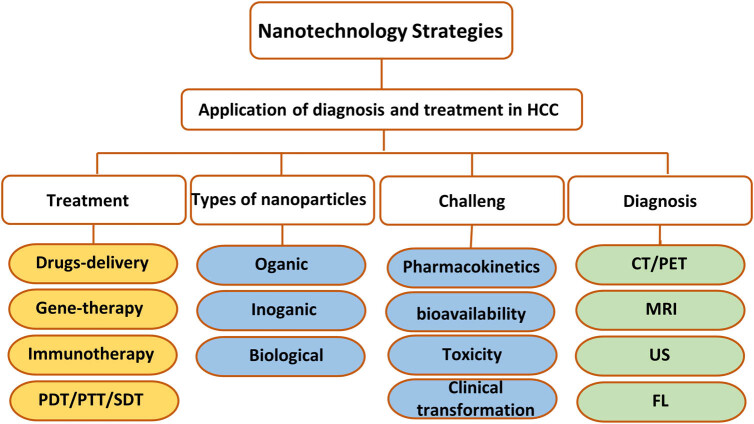
Nanotechnology strategies for HCC.

Given the inadequacies of current diagnostic and therapeutic approaches for liver cancer, there is an urgent need for new technologies and drugs to enhance the precision treatment of HCC and improve patient outcomes. Nanotechnology offers an alternative to conventional medicine. Today, there are more than 100 types of cancer in which nanoparticles (NPs) have shown good therapeutic effects against lung, pancreatic, lymphatic, and brain tumors, which means they have properties that allow them to penetrate cancer cells. NPs can overcome the tumor interstitial barrier and, through binding to targeted substances like antibodies, peptides, and small molecules, deliver drugs specifically to tumor tissues without causing the adverse effects associated with traditional anti-tumor agents, and prevent drug molecule degradation [5,10]. Various NPs are being developed for use, including polymer NPs, gold nanoparticles (AuNP), silver NPs, silica NPs, magnetic NPs, and lipid NPs [11]. These NPs, combined with anti-tumor drugs, exploit their physical properties for imaging as contrast agents and respond to external stimuli such as sound, light, electricity, magnetism, and heat to integrate drug therapy with physical treatments like sonodynamic and photothermal therapy (PTT) for HCC, achieving integrated diagnosis and treatment. Integrated treatment combines diagnosis and therapy into one, integrating multiple components with tumor diagnostic and therapeutic functions onto a single NP platform, offering a multifunctional NP diagnostic and therapeutic agent capable of early tumor diagnosis and precision treatment, while also enabling real-time monitoring of treatment effects and prognosis. Since 1998, when the term “theranostics” was first introduced by pharmacology CEO John Funkhouser, it has been widely applied in the biomedical field as a new personalized diagnostic and therapeutic strategy within precision medicine aimed at monitoring treatment responses, enhancing drug efficacy and safety, and eliminating unnecessary treatments, thus saving significant healthcare system costs [12]. This article reviews the latest research progress in the diagnosis and treatment of liver cancer, the types of nanomaterials, and their application to HCC-targeted imaging, therapy, and integrated treatment, discussing the opportunities and challenges of new nanomaterials in targeted imaging and treatment of liver cancer (Figure 1).

## 2 Mechanisms of action of nanomaterials in liver cancer

### 2.1 Nanomaterials for targeting and drug delivery

**Figure 2:**
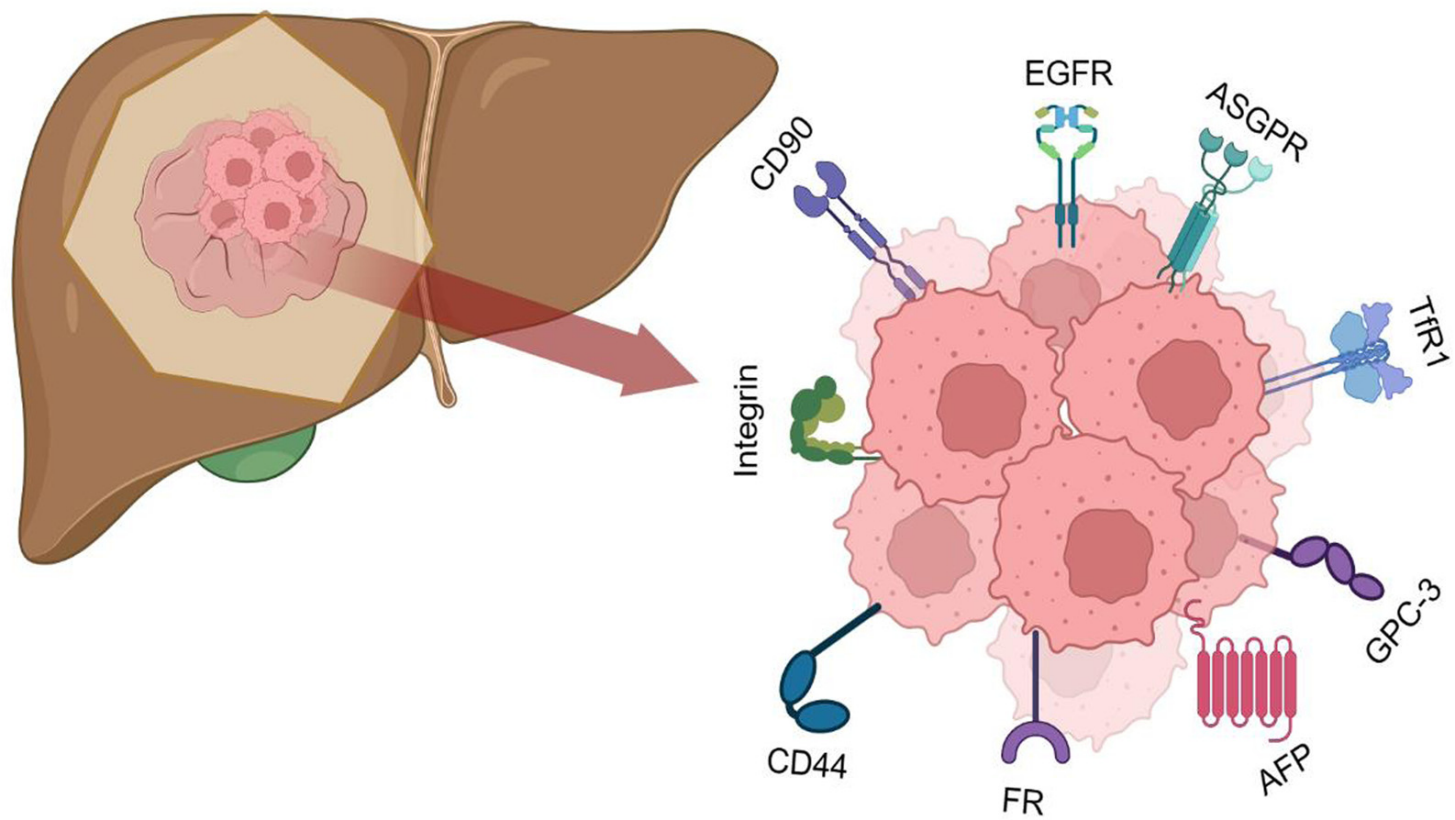
The above is a list of the receptors that are overexpressed in HCC cells.

Traditional liver cancer therapies often lack the capability to distinguish between cancerous and healthy cells, leading to ineffective treatments and systemic adverse reactions due to the uptake of drugs by normal liver cells. The emergence of nanotechnology holds vast potential for liver cancer treatment. NPs possess a large surface area to volume ratio, high stability, small size, high biocompatibility, and unique optical, acoustic, electric, and magnetic properties, making them ideally suited for drug absorption and subsequent controlled release [13]. Research indicates that NPs approximately 60 nm in diameter are particularly efficient at accumulating in liver cancer tissues [14]. NPs can enter liver cancer tissues through pores and the lymphatic system. The liver’s reticuloendothelial system, composed of Kupffer cells, preferentially absorbs negatively charged particles, leading to liver toxicity, whereas hydrophilic groups help evade the liver’s barrier mechanisms [15]. The rich blood supply and large exchange surface area within liver sinusoids result in slow blood flow, facilitating the deposition of NPs in the liver [4]. Large molecules and colloidal systems of NPs can selectively localize in solid tumors via a passive mechanism known as the enhanced permeability and retention (EPR) effect. The pathological basis for passive targeting is an abnormal vascular system, but this method can be ineffective due to the unique anatomical and pathological features of tumor tissues, which prevent the nanoconjugates loaded with targeted drugs from entering tumor tissues [16]. In contrast, actively targeted nanomedicines, after surface modification and through specific interactions, internalize into cells via receptor-mediated endocytosis, a common strategy for actively enhancing targeting in HCC [5,17]. Many receptors are overexpressed on the surface of liver cancer cells but are absent or minimally present on normal liver cells, making receptors such as asialoglycoprotein receptor (ASGPR), glypican-3 (GPC-3), transferrin receptor (TfR), folate receptor (FR), AF-20 antigen, somatostatin receptor, and cluster of differentiation 44 increasingly exploited for specific HCC targeting (Figure 2) [18,19,20,21,22,23]. Many drugs aim for better targeting to liver cancer tissues by modifying the surface of drug-loaded NPs with peptides, small molecules, antibodies, vitamins, and other biological elements [24,25]. The multifunctional modification of nanocarriers has become a hot research topic in recent years. Chi et al. [26] constructed NP carriers loaded with mesoporous silica NPs containing arsenic trioxide prodrug (NiAsO_*x*_), surface-modified with the targeting ligand folic acid (FA) to enhance therapeutic efficacy. Huang et al. designed exosomes with GPC3 Single-chain variable fragment (scFv) antibodies on their surface using the pDisplay vector and loaded them with IR780 and lenvatinib. The results showed that these engineered exosomes (IR780@GPC3-EXOs) rapidly targeted HCC and significantly inhibited liver cancer cells through photothermal effect and lenvatinib’s action post-near-infrared (NIR) stimulation [27]. Researchers have shown that incorporating cell membranes into NPs can improve their accumulation in cancer tissue. NP wraps around a membrane obtained from the patient’s own cancer cells and will usually attach to the patient-derived cancer cell line. NPs wrapped around macrophages or white cell membranes can recognize tumors, while hybrid membranes, such as red blood-cancer cell hybrid membranes, can further improve specificity. NP using these membranes showed a two- to threefold increase in drug activity over free drugs [28]. Nanotechnology utilizes various NP-based drug delivery systems to reduce the amount of drug needed to improve the therapeutic index, minimize systemic toxicity, extend drug release after a single dose, and enhance selective targeting of liver cancer cells. These drugs can be chemotherapeutic agents, natural plant medicines, and gene therapy drugs.

#### 2.1.1 Delivery of molecularly targeted chemotherapy drugs

Nanotechnology facilitates the encapsulation of chemotherapy drugs into various NPs, creating functionalized NPs with enhanced targeting and efficacy. This increases the drug concentration at tumor sites, prolongs local blood drug concentration, and reduces systemic toxicity and adverse effects. NPs overcome the limitations of traditional chemotherapy, showing immense potential for precision cancer therapy [29]. Sorafenib and Lenvatinib, frontline chemotherapy drugs for HCC, are recommended globally for advanced HCC treatment. Although the average survival time of patients increased by only 3–5 months compared to the placebo group, the results were far less than ideal [3,30,31], with long-term use leading to resistance and adverse reactions such as increased serum lipase and amylase concentrations, hypertension, neuropathy, leukopenia, lymphopenia, diarrhea, nausea, vomiting, and respiratory difficulties [32], researchers are increasingly focusing on loading chemotherapy drugs onto NPs for precise drug delivery, enhancing efficacy and reducing toxic effects. For instance, Albalawi et al. [33] developed a sorafenib chitosan NP delivery platform using ionotropic gelation, showing that at the same time point, the anticancer effect of sorafenib-loaded NPs was more effective than free drug and non-toxic to normal adult human skin fibroblast (HDFa) cell lines. Sorafenib-loaded NPs exhibited high release efficiency, bioavailability, improved solubility, and targeted tumor tissues actively [34,35].

#### 2.1.2 Delivery of natural plant medicines

**Figure 3:**
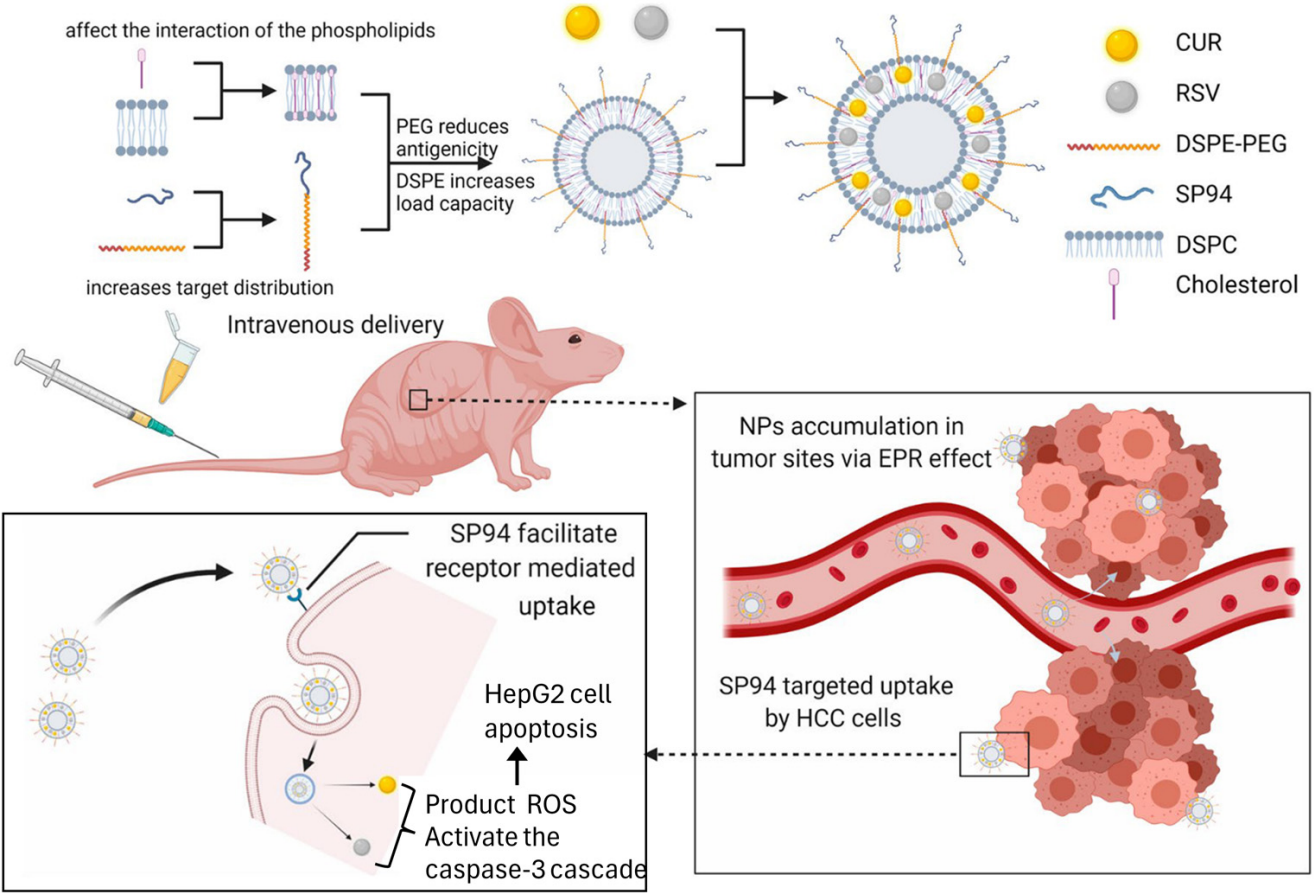
Schematic diagram of the mechanism of action of NPs (SP94-NP-CUR + RSV) on HCC. By 189 preparing NPs, which were surface modified with ligand SP94 and loaded with CUR and RSV and injecting it through the tail vein of mice, the NPs accumulate at the tumor site through the EPR effect. 191 Meanwhile, due to the targeting effect of SP94, the NPS accumulates at the tumor site. NP is specifically taken up 192 by HCC cells, and after endocytosis into HCC cells, CUR and RSV are released from the NPs, promoting 193 ROS production and activating the caspase-3 cascade reaction, leading to apoptosis of HepG2 cells [42]. (Open Access) Copyright from BioMed Central.

In recent years, components from natural plants have been increasingly used for liver cancer treatment, offering low toxicity and easy accessibility. Medicinal plants contain many new key bioactive substances that act as blocking agents or reducing agents during NP synthesis, which can inhibit the activation of carcinogenic pathways at the cellular level and effectively fight different types of cancer, such as quercetin, curcumin (CUR), resveratrol (RSV), epigallocatechin-3-gallate, and other molecules have been widely studied. Bioactive substances extracted from plants have high efficiency, minimal toxicity, and the ability to overcome drug resistance [36,37]. However, their poor water solubility, low cell uptake, and low bioavailability limit their widespread application, which can be addressed by NP delivery systems. Nanotechnology-based approaches, or nanomedicines, can provide pathways to bypass limitations associated with plant bioactivity and help increase bioavailability, improve cell uptake through site-specific targeting, and achieve stable concentrations of bioactivity throughout the therapeutic regimen [38,39]. CUR, a natural polyphenol extracted from turmeric, exhibits low toxicity, anti-inflammatory, and anticancer properties and can enhance liver cancer cells’ sensitivity to chemotherapy drugs. Cheng et al. [40] used an inverse microemulsion and thin-film dispersion method to prepare a liposome loaded with cisplatin and CUR (CDDP/CUR-Lip) for liver cancer treatment, finding that the combined effect of CUR and cisplatin loaded on a lipid NP platform not only reduced the toxicity induced by cisplatin but also enhanced the anticancer effect of the chemotherapy drug. Piperine, an alkaloid extracted from black and long pepper, exhibits anticarcinogenic, antiproliferative, and antioxidant properties against various types of cancer, including HCC [41]. Zheng et al. designed injectable NPs for the specific targeted delivery of CUR and RSV to liver cancer cells. The NPs, modified with HCC-specific peptide SP94, exhibited high permeability and the EPR effect, prolonging the action time of CUR and RSV in tumor tissue, significantly reducing the dose required, delaying drug release rate, and enhancing the bioavailability of the encapsulated drugs [42] (Figure 3). In addition, some NPs can even be isolated directly from plants, vesicular NPs (PDVLns) being the general term for vesicular nanostructured particles isolated from plants. PDVLN is a natural nanocarrier containing lipids, proteins, DNA, and microRNA (miRNA) that can enter mammalian cells and regulate cell activity. PDVLN has great potential in immune regulation of macrophages, regulation of gut microbes and friendly antioxidant activity, as well as overcoming drug resistance, and it has shown some therapeutic efficacy in inflammatory bowel disease and colitis-related cancers. Its low immunogenicity and wide availability make PDVLN safer and more economical to develop as a therapeutic agent and drug carrier [43,44].

#### 2.1.3 NP-mediated nucleic acid delivery

Gene therapy involves targeted regulation of gene expression within specific cells, utilizing pathways of apoptosis, cell cycle checkpoints, and RTK signaling to modulate cell death, proliferation, and survival, offering a promising and effective cancer treatment method. Introducing exogenous nucleic acids such as DNA, mRNA, short interfering RNA (siRNA), miRNA, or antisense oligonucleotides can correct or ameliorate tumor symptoms [45,46]. These can be delivered to tumor tissues via recombinant viruses (also known as biological NPs or viral vectors) and non-viral methods. In human liver cancer cell line Huh7, overexpression of the GPC3 gene was noted. Lei et al. successfully constructed a recombinant plasmid (PGC-shRNA-GPC3) transfected into the Huh-7 liver cancer cell line, significantly inhibiting GPC3 mRNA expression levels [47]. scFv modified exosomes targeting GPC 3 loaded with miR-26a were effectively delivered to GPC 3 positive HCC cells, thereby inhibiting the proliferation and migration of liver cancer cells through the regulation of miR-26a downstream target gene expression [48].

### 2.2 Integration of nanotherapy with other technologies for liver cancer treatment

#### 2.2.1 NPs combined with phototherapy (PDT/PTT) for liver cancer treatment

Compared to traditional chemotherapy, PTT offers the advantages of minimal invasiveness, high selectivity, and lesser cosmetic damage to patients, making it a fast, precise, and cost-effective cancer treatment approach [49]. PTT and photodynamic therapy (PDT) are both PTT methods. PTT uses photothermal converters to transform light energy into heat [50], ablating local tumors, and is often used for early-stage, single tumors (diameter less than 2 cm). PDT induces tumor cell apoptosis through the generation of cytotoxic reactive oxygen species (ROS) [51].

Evidence suggests that PDT and PTT can have synergistic effects, with the combination of PDT/PTT gaining momentum due to the development of multimodal NP platforms combining photodynamic and photothermal agents. This combination can overcome the limitations of single treatment modalities. Dual-modality multifunctional photoactive NPs suitable for both PDT and PTT not only increases tissue heating but also generates ROS. Inorganic materials like gold and metal oxide NPs, silica, upconversion NPs (UCNPs), and quantum dots (QDs) are characterized by high photothermal conversion coefficients and can be functionalized with photosensitizers [52]. Modifying NPs with targeting ligands can also enhance the selectivity of PDT/PTT toward tumor tissues [53]. Hu et al. [54] developed a NP composite (UCNPs@mSiO2-Ce6-GPC3) internally loaded with UCNPs excited by 808 nm NIR light and photosensitizer dihydroporphyrin e6 (Ce6), further modified with GPC-3 targeting ligand for liver cancer, effectively inhibiting HepG2 cancer cell growth with good biocompatibility and low toxicity. Since PTT does not depend on the local oxygen level in tumor tissues, it can induce additional cell death after local oxygen levels are depleted following PDT. It has been found that performing PTT (808 nm) after PDT (655 nm) using gold nanorod-dihydroporphyrin e6 NP gel does not improve tumor growth compared to the PTT group alone. However, performing PTT after PDT can significantly inhibit tumor growth [55].

#### 2.2.2 NPs combined with sonodynamic therapy (SDT) for liver cancer treatment

SDT is a non-invasive treatment method with sufficient tissue penetration depth, proven to be effective for liver cancer treatment. It offers high treatment efficiency, low side effects, and low cost, making SDT combined with NPs a novel treatment approach that somewhat compensates for the poor penetration depth of PDT. The treatment mechanism of SDT primarily involves sonocavitation effects and the production of highly toxic ROS, inducing necrosis or apoptosis of liver cancer cells. It can also form tumor-specific immunity by damaging blood vessels or inhibiting angiogenesis in tumor tissues [56]. Nanobubbles loaded with the sonosensitizer dihydro porphyrin (Ce6) and miR-195 through SDT and gene expression regulation trigger immunogenic cell death, thus eliciting a strong immune response against liver cancer [57]. Janus Au-MnO NPs (JNPs) responsive to ultrasound (US) and glutathione (GSH) disassemble into smaller Janus Au-MnO NPs post-SDT, then respond to high levels of GSH in the tumor area to further disintegrate into smaller Au NPs and Mn^2+^ ions, enhancing NP penetration ability and further boosting the SDT cavitation effect and ROS production to inhibit the growth of liver cancer cells [58].

### 2.3 Nanomaterials for immunotherapy

Immunotherapy, contrary to conventional anticancer agents that directly exhibit cytotoxicity, activates immune cells to recognize and eradicate tumor cells. Although immunotherapy holds broad prospects in cancer treatment, its efficacy is limited by the immunosuppressive tumor microenvironment and systemic toxicity, hindering the widespread application of cancer immunotherapy [59]. Combination immunotherapy methods, improving tumor selectivity, can enhance anticancer efficacy while avoiding systemic toxicity. Tumor-targeting lipid dendrimer-calcium phosphate NPs functionalized with thymidine kinase deliver siRNA against the immune checkpoint ligand PD-L1 and the immunostimulatory IL-2 encoding plasmid DNA to HCC, increasing tumor infiltration and CD8+ T cell activation, enhancing the efficacy of cancer vaccine immunotherapy and inhibiting HCC progression [60]. Guo et al. [61] developed a nanoprecipitation technique to produce a nanomedicine containing oxaliplatin derivatives and folinic acid (termed Nano-Folox) as well as a nanomedicine containing FdUMP (active metabolite of 5-Fu), which when combined in an HCC mouse model, achieved synergistic effects due to nano-FdUMP-mediated ROS formation, thus promoting the efficacy of nano-Folox-induced immunogenic cell death.

It is worth noting that most anti-tumor nanomaterials mainly rely on the production of ROS to promote apoptosis, while ROS is more dependent on oxygen molecules at the tumor site. Therefore, improving the oxygen-poor microenvironment of tumors and increasing the ROS level in cancer cells has always been considered an effective strategy to eradicate cancer cells, and most ROS comes from the mitochondrial respiratory chain. Among the many antioxidants and detoxifying enzymes present in mitochondria, mitochondrial GSH is the main line of defense in maintaining an appropriate mitochondrial REDOX environment to avoid or repair oxidative modifications that lead to mitochondrial dysfunction and cell death [62]. GSH is an important member of the intracellular antioxidant system. Its antioxidant function is mediated through two pathways: Elimination of free radicals by reacting with ROS, active nitrogen, hydroxyl radicals, hypochlorous acid, and other active species, or as an indispensable cofactor of many enzymes, is considered the most abundant molecule of endogenous antioxidants in cancer cells, and high levels of GSH are essential for the removal of excess ROS and detoxification of foreign substances. This makes it a potential target for cancer treatment. Numerous studies have shown that the loss of intracellular GSH makes cancer cells more vulnerable to oxidative stress and chemotherapy drugs. GSH depletion has been shown to improve the efficacy of ROs-based therapies (photodynamic therapy, SDT, and chemokinetics), ferroptosis, and chemotherapy [63,64].

Therapeutic nanocarriers delivery agents for HCC are listed in Table S1 – you can see this form in the supplement file, reference [18,20,33,40,47,65–83].

## 3 Types of nanomaterials and their applications in liver cancer diagnosis and treatment

### 3.1 Inorganic NPs

Inorganic NPs include QDs, metal oxide NPs, AuNPs, silver NPs, nanodiamonds (ND), calcium NPs, and nanofibers. QDs, AuNPs, metal oxide NPs, and carbon-based nanomaterials are most commonly used, primarily as drug carriers, with the tumor microenvironment serving as the main stimulus-response mechanism for constructing drug release systems targeting both therapy and diagnosis in liver cancer [84].

#### 3.1.1 Metal NPs

Metal NPs are modified into various forms for distinct applications due to their unique physical and chemical properties, among which gold and silver NPs are most commonly used because of their biocompatibility.

AuNPs are especially notable for their adjustable size and surface properties, playing a significant role in drug delivery due to their stability, ease of surface functionalization, and selective targeting capabilities. As one of the least toxic metal NPs, AuNPs primarily serve as carriers for the precise delivery of chemotherapy and gene therapy drugs to HCC tissues. Furthermore, they can act as photothermal agents, leveraging photothermal ablation to inhibit the growth of liver cancer cells [85,86]. The tumor-suppressing miRNA, miR-375, which is downregulated in HCC, can be effectively delivered to HCC cells via AuNP-miR-375 NPs; this results in significant uptake by liver cancer cells, inhibiting tumor cell proliferation, migration/invasion, colony formation, and inducing apoptosis [87]. The combination of AuNPs loaded with gene therapy drugs and chemotherapy drugs like sorafenib can more effectively inhibit the proliferation of HCC cell lines than either treatment alone [65].

Silver NPs, prepared similar to AuNPs, also exhibit biocompatibility, solubility, and stability [5]. They differ from AuNPs in their mechanism of action in liver cancer tissues, primarily inducing liver cancer cell apoptosis through ROS-dependent pathways and oxidative stress, demonstrating anticancer effects against HCC. Studies have shown that the cytotoxic effects of silver NPs on HepG2 cell line and primary hepatocytes are mainly achieved by promoting ROS production, inhibiting GSH reduction, and leading to membrane oxidation, protein carboxylation, and DNA damage. Silver NPs can also change the normal function of vascular endothelial factors, further validating AgNPs as potential cytotoxic drugs and candidates for anticancer therapy [68,88,89].

#### 3.1.2 Magnetite NPs

Magnetic nanosystems come in various types, including magnetic nanofibers, multifunctional magnetic NPs, magnetic nanoclusters, magnetic hollow mesoporous silica nanospheres, superparamagnetic iron oxide NPs (SPIONs), and QDs [90]. SPIONs and QDs are the most widely applied magnetic nanosystems due to their high magnetic saturation, low toxicity, optical and fluorescent properties, and stability in biological fluids, often used in targeted drug delivery and PTT for liver cancer, integrating diagnosis and therapy [90]. The efficacy of MNPs as drug carriers increases with biocompatibility enhancements. They are encapsulated by liposomes, proteins, polysaccharides, and other biomolecules, also facilitating precancerous liver cancer treatment [91]. Surface-modified folate-targeted arsenite-loaded magnetic mesoporous silica NPs integrate imaging and therapy, offering significant hope for HCC treatment [26].

#### 3.1.3 Selenium NPs (SeNPs)

Selenium is a trace mineral essential for the maintenance of various processes in the body [92], which can maintain immunoendocrine, metabolic, and cellular homeostasis. In recent years, the research on selenium has been deepening, and some scholars have found that low doses of selenium show antioxidant properties, and large doses show pro-oxidation properties. Conversely, low concentrations of selenium protect healthy cells and tumor cells, support DNA repair, and relatively high concentrations of selenium can be used for anticancer treatment and reduce the risk of carcinogenesis and various cell mutations. In addition, selenium inhibits the migration of tumor cells, meaning that it prevents the development of tumor metastases, an effect that has been demonstrated in cases of breast, prostate, colon, lung, and lymph node metastasis [93]. Recent clinical trials have shown that selenium yeast supplementation is effective in reducing the incidence of prostate cancer by about 60% and is able to reduce the overall mortality rate of colorectal and lung cancer by about 50% [94]. Although selenium shows significant anticancer effects, its anticancer mechanism is less well understood, mainly depending on the form, dose, time of action of selenium, and the characteristics of tumor cells. High doses of selenium produce oxygen free radicals, which initiate the apoptosis mechanism of cancer cells by participating in the process of protein conformational structure (signaling molecules, inhibitory enzymes and transcription factors) changes. Although selenium has shown significant anticancer effects, it is impossible to avoid excessive selenium intake, which can lead to toxicity, psychiatric problems, and cancer [95]. SeNPs are considered to be a promising drug delivery system with the advantages of anti-tumor activity, reduced cytotoxicity, and high drug loading [96]. It was found that BerSenps and Ber-AgNPs prepared by berberine (Ber), silver, and selenium up-regulated the activities of intracellular P53, Bax, cytochrome C, and caspase-3 and down-regulated the level of BC-L-2, thus playing a good anti-liver cancer effect, and the cell mobility was significantly reduced compared with the control group. In addition, Ber-SeNPs showed better therapeutic outcomes than Ber-AgNPs, which may benefit from the inherent anticancer properties of selenium [97].

### 3.2 Organic NPs

Organic NPs encompass dendrimers, emulsions, aptamers, solid lipid NPs, nanobodies, and other polymers. Among these, the first Food and Drug Administration (FDA)-approved nanodrug was based on liposomal nanocarriers, while poly(lactic-*co*-glycolic acid) (PLGA) NPs and polyethylene glycol (PEG) NPs are also FDA-approved organic NPs commonly used in the treatment of HCC [98,99].

#### 3.2.1 Liposomes

Liposomes are vesicular drug delivery systems composed of a phospholipid core and a lipophilic lipid bilayer, capable of encapsulating both hydrophilic and hydrophobic drugs. By targeting drug delivery, they enhance bioavailability and reduce drug toxicity, making them widely used for encapsulating both hydrophilic and lipophilic drug molecules. They are one of the most common carriers for targeted drug delivery in HCC, with many liposome-based drug formulations already on the market [100,101]. Besides encapsulating chemotherapy drugs for HCC, they can also carry gene therapy drugs such as doxorubicin, sorafenib, cisplatin, and siRNA [40,78], with surface modifications for HCC-specific targeting ligands commonly targeting ASGPR, heparan sulfate proteoglycans, and FR [102]. Liposomes are also convenient for encapsulation and delivery of plant crude extracts to tumor sites. When RSV is encapsulated in cationic liposomes, its uptake rate is not only higher than that of RSV alone, but also its bioavailability is increased by 3.2 times. At the same time, the localization of the drug in tumor tissues is increased [103].

#### 3.2.2 Solid NPs

Solid NPs are colloidal systems primarily composed of heavy lipids, offering a solution for insoluble drugs in liver cancer treatment. They possess excellent biocompatibility, high drug-loading capacity, long-term stability, and feasibility for large-scale production, making them of particular interest as oral drug delivery carriers [7]. Studies have shown that solid NPs can enhance the intracellular delivery of anticancer drugs. The combination of ganoderic acid (GA) with solid lipid NPs (GASLNs) demonstrated higher cytotoxicity against liver cancer cells than GA solution and blank SLNs, significantly reducing the size of liver cancer nodules and altering oxidative stress levels in a dose-dependent manner [7]. Modifying solid NPs with liver cancer-targeting receptor ligands can further enhance the bioavailability of drugs. Boronate-modified solid lipid NPs (Gal-SLN/BTZ) carrying bortezomib (BTZ) delivered BTZ to HCC cells, where *N*-stearoyl lactobionic amide (N-SALB) targeted NPs exhibited greater cytotoxicity against HepG2 cells and achieved the highest percentage of cell apoptosis compared to non-targeted NPs [18].

#### 3.2.3 Carbon-based nanomaterials

Carbon-based nanomaterials encompass various forms, such as graphene (GRP), carbon dots, NDs, carbon nanotubes, fullerenes (FUL), and QDs. Due to their toxicity to humans and the environment, their applications in liver cancer have primarily focused on developing sensors for cancer biomarkers [104]. Recently, these nanomaterials have been increasingly utilized for targeted drug delivery, cancer diagnosis, and PTT in treating liver cancer. GRP QDs, serving as photothermal agents, have led to the development of an aptamer-modified GRP QD/magnetic chitosan direct drug delivery system for liver cancer photothermal chemotherapy, significantly inhibiting tumor growth and extending the survival of tumor-bearing mice [105].

#### 3.2.4 Polymer micelles

To date, different colloidal drug delivery systems (nanosystems) based on various polymers have been designed and explored to produce carriers with physicochemical and biopharmaceutical properties that improve HCC treatment [106]. Polymer micelles can be obtained from a variety of polymers, with the most common being PEG NPs and PLGA NPs. These NPs, composed of self-assembled amphiphilic block copolymers, form a hydrophobic core and a hydrophilic corona. In 1975, Duncan proposed a macromolecular prodrug model, which consisted of multiple drug molecules linked to a macromolecule, i.e., multifunctional polymers [107]. Drugs or proteins conjugated to polymers and polymer micelles formed by the self-assembly of drug-polymer monomers are known as “polymer therapeutics.” Polymer micelles have been used to treat HCC, encapsulating hydrophobic drugs like ursolic acid, chloroxine, and quercetin to increase their bioavailability [80,108,109].

### 3.3 Biological NPs

Biosynthetic NPs are a simple, affordable, environmentally friendly, and highly feasible method that offers a promising alternative to the currently widely used physical and chemical methods [110]. Biological NPs are mainly composed of biological molecules (such as proteins, nucleic acids, sugars, etc.) or nanoscale particles synthesized by biological processes. According to their different sources, composition materials, or functions, they can be divided into natural biological NPs (such as viruses, liposomes, etc.) and synthetic biological NPs (such as DNA-based nanostructures, protein-based NPs, etc.) [111]. The preparation and packaging process of protein NPs does not use toxic chemicals or organic solvents and has many advantages, such as safety and reliability, biodegradability, and biocompatibility [111,112,113]. Wong et al. [114] prepared a protein NP assembled from a fusion protein consisting of an amphiphilic helical peptide of M2 protein derived from H5 N1 influenza virus (AH3) and a superfolded green fluorescent protein (sfGFP), creating a mutant with two gain-of-function mutations. Contributes to the higher thermal stability of protein NPs and stimulates a durable humoral immune response after a single immunization.

**Figure 4:**
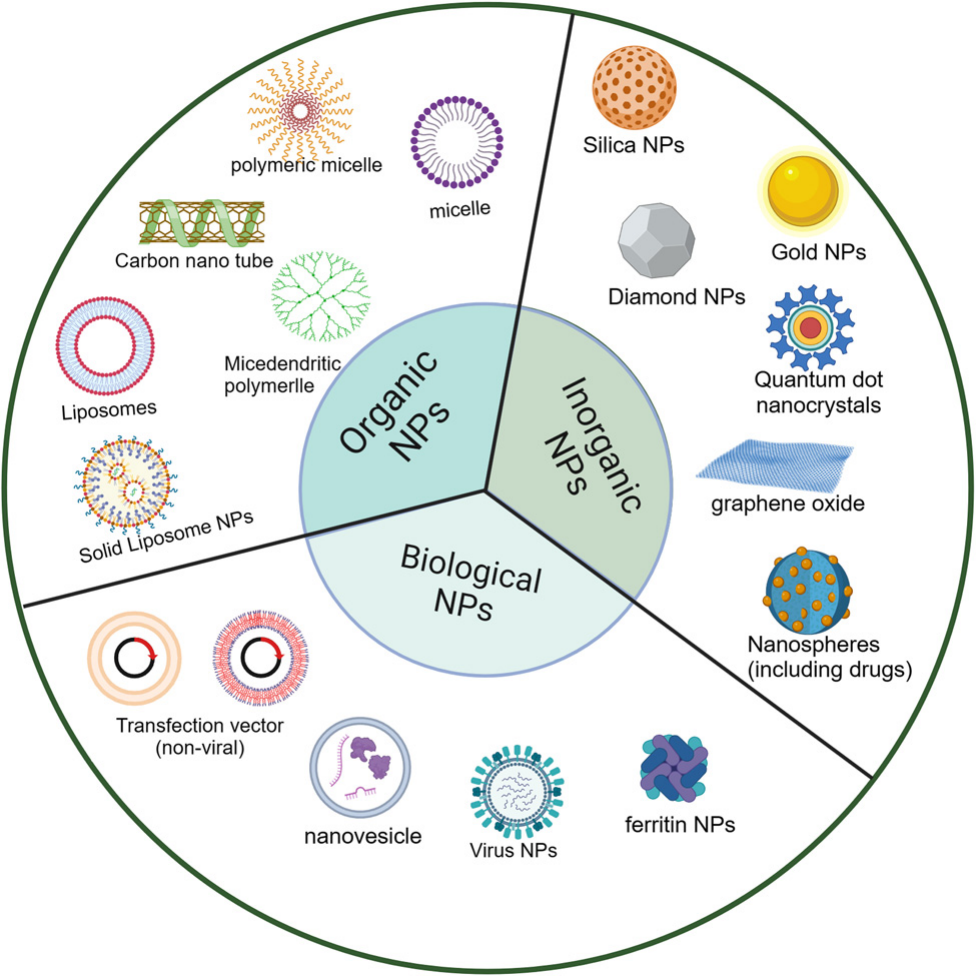
Different types of nanomaterials used in nanomedicine (created with BioRender.com).

In addition, using programmable self-assembly capabilities, DNA is considered a powerful building material for creating nanostructures with specific shapes and functions, and due to its high degree of biocompatibility, DNA molecules can be safely used in natural biological processes. Many DNA nanostructures with different structures and functions have been reported for the reengineering of natural systems [115,116,117]. We introduce the various types of nanocarriers currently available, including organic, inorganic, and biological NPs (Figure 4).

## 4 Challenges in the application of nanomaterials in HCC

As nanotechnology becomes more and more mature, more nanomaterials are gradually developed and tried for biomedical applications, however, due to some unavoidable challenges, such as lack of effective drug delivery routes, low bioavailability, channels across biological barriers, degradation, and toxicity of nanomaterials. As a result, most nanomedicine-based materials only stay in the *in vivo* and *in vitro* stage; clinical transformation is difficult, and only a small part of them really enter clinical trials.

### 4.1 Pharmacokinetics, bioavailability, and distribution of NPs

The physical and chemical properties of NPs, including their size, morphology, surface charge, and surface chemical treatment (e.g., pegylation, ligand coupling) and composition, can significantly affect the kinetic behavior of drugs *in vivo*, their distribution *in vivo*, their penetration in tumor tissues, and the bioavailability of tumor cells. Each class of NPs exhibits unique properties as well as advantages and disadvantages of the characteristics (Table S2, you can see this form in the supplement file, reference [8,100,106,118–133]). Due to the EPR effect, when the size of the NP is less than 5.5 nm, it is mainly filtered through the kidney, while when it is less than 50 nm, it interacts with liver cells and is metabolized from the liver [134]. During intravenous administration, NPs usually accumulate in the capillary bed, resulting in premature release of nanotherapeutic drugs at this site, leading to significant toxicity. When NPs are smaller than 20 nm, they usually accumulate more at the tumor site, but they are also rapidly filtered and excreted, resulting in reduced cycle time. However, when the size of NPs is greater than 200 nm, the complement system is activated and rapidly expelled from the circulation. The distribution of NPs at tumor sites is uneven, and the main reason affecting their biological distribution is the presence of uncomplexed free cationic polymers in the cationic formulation. Some scholars have demonstrated that liver GSH-mediated biotransformation can be used to regulate the biological interaction of NPs in order to maximize tumor targeting and reduce the accumulation of NPs in other sites [135].

### 4.2 Toxicity of NPs

Although NPs are composed of biological materials with high safety, they are of major medical and health concern due to the potential risks of these materials to tissues, cells, and organs, which can interact with the human body and cause unexpected and dangerous toxicity [84]. The toxic mechanisms of nanomaterials include inflammation, oxidative stress, apoptosis, necrosis, and genotoxicity [136,137,138]. The surface area, particle size, shape, solubility, and agglomeration of NPs are all factors contributing to their toxicity. Due to the very small size and wide surface area of NPs, they may also affect genotoxicity. ROS generated by inflammation and oxidative stress can interact with DNA and eventually lead to DNA oxidation and breakage. However, genotoxicity can be detected *in vitro* by the Ames test, Comet test, and chromosome aberration test, as well as *in vivo* (rodent carcinogenicity, chromosome aberration, and endogenous gene mutation) [139]. Studies have shown that NPs can be deposited in the lung, thus causing inflammation, oxidation, and cytotoxic effects [140]. The size and shape of NPs affect their distribution, deposition, and clearance in the kidney and liver. Meanwhile, charged NPs have higher accumulation in target organs than uncharged NPs. After oral or intravenous administration, NPs in ionic form accumulated more in organs such as liver, lung, and kidney [141], and silver NPs with a diameter of 10 nm had higher tissue distribution and more severe hepatotoxic reactions [142]. Some scholars have proposed that the surface properties of NPs can be modified by using different coating materials to reduce potential toxicity, such as PEG, which can reduce toxicity by changing the interaction between NPs and proteins.

### 4.3 Clinical transformation of nanomaterials

Despite tremendous technological advances in tumor nanotherapeutics, the complexity and heterogeneity of tumor biology, as well as the lack of a comprehensive understanding of nanobiotic interactions, remain important barriers to future clinical translation and commercialization. Currently, liposomes, polymer NPs, SiRNA-bound lip-based NPs, mirnas, and polymer micellar NP systems have entered clinical trials and been approved by the U.S. FDA [143]. Doxorubicin is the first anticancer nanocide approved for clinical trials [144]. Although the nanocide has been effectively approved for clinical trials, if disease progression or drug resistance occurs during clinical trials, the clinical trial results will be distorted and the nanocide will be prevented from continuing treatment. In response to this adverse situation, some scholars have synthesized a HCC targeting liposome HCSP4/Lipo-DOX/miR125a5p that inhibits doxorubicin delivery with multi-drug resistance. The results show that the liposome has specificity and sensitivity to liver cancer cells, and has satisfactory therapeutic effect on HCC, especially drug-resistant HCC [145]. The technical challenges regarding the clinical application of NPs are mainly focused on three aspects: equilibrium optimization, amplification of the synthesis process, and performance prediction. In applications involving *in vivo* and *in vitro* studies, NPs are mostly manufactured through small-scale production methods. This is mainly limited by equipment conditions and a variety of other factors, so significantly expanding these production scales is not always a realistic option. Some scholars have extensively tested a variety of nanoformulations, and with the help of a precise selective iterative process, microfluidic technology has become a powerful tool to overcome this challenge by synthesizing a large number of nanoformulations with improved particle properties and product purity [146,147]. In addition, most studies are carried out around “cell and animal models,” but these models are relatively simple, so it is difficult to simulate the natural response of the human body. If we focus on personalized medicine, *N* = 1 clinical study will be required, which must consider many factors, such as genetics, environment and past medical history, and the workload is large, resulting in the difficulty of clinical trials [100]. However, with the unremitting efforts of researchers, the clinical progress of NPs is still continuing, and at present, PIHCA polymer NPs have achieved considerable efficacy in Phase III clinical trials for the treatment of advanced liver cancer [148].

## 5 Nanomaterials in liver cancer diagnostic applications

### 5.1 Integration of nanotechnology with imaging diagnostic techniques

Contrary to most solid cancers, HCC does not always require histological confirmation. Non-invasive diagnosis is possible and relies on imaging examinations [149]. Biannual US monitoring of patients who evolve from cirrhosis to HCC allows diagnosis at an early stage when effective treatment is feasible, so two-dimensional US imaging is a valuable tool for monitoring patients at risk for early HCC, while CT and MRI are common diagnostic methods for HCC [150,151].

#### 5.1.1 Nanotechnology combined with US imaging for liver cancer diagnosis

US is an effective, inexpensive, and non-invasive method for monitoring early-stage liver cancer. However, two-dimensional US often fails to clearly delineate necrotic areas. Thus, the development of US contrast agents (UCA) capable of differentiating liver cancer from normal liver tissue has simplified and enhanced the diagnosis of HCC with advanced US imaging techniques [4]. Doxorubicin NP microbubbles (Dox-NP-MB) used in an in situ rat liver cancer model, observed with enhanced US, demonstrated the therapeutic effects of Dox-NP-MB on rat liver cancer [152]. Nanobubbles loaded with GRP oxide, transforming ultrasonic energy into heat via ultrasonic cavitation effect, enhance ablation and tissue penetration capabilities, serving as UCAs, playing a crucial role in the early monitoring of liver cancer [4]. Researchers have applied NP-enhanced transmission US (NESUS) as an image-based monitoring mode for microwave thermotherapy treatment in breast cancer [153]. UCA are functionalized by coupling ligands to their surface to target specific biomarkers of diseases or pathological processes [154].

#### 5.1.2 Nanotechnology combined with CT/MRI imaging techniques for liver cancer diagnosis

Recent developments in nanomedicine have continuously enhanced the accuracy of CT and MRI imaging for liver cancer diagnosis, with an increasing number of efficient contrast agents being developed. Liu et al. prepared chitosan (CTS), triphosphate (TPP) NPs, polyacrylic acid (PAA) further conjugated with cysteine-functionalized AuNPs, termed CTS/TPP/PAA@AuNPs (CTPA), using a mouse liver cancer model, showing that multifunctional CTPA can achieve effective drug delivery and CT imaging [155]. The cost of AuNPs might be higher than iodine-based contrast agents, especially in developing countries, limiting the use of AuNPs for CT diagnosis of HCC.

In the field of MRI imaging, gadolinium, a traditional MRI contrast agent, is not specific in distribution within the body, has low sensitivity for early-stage liver cancer, and possesses some degree of nephrotoxicity. The advent of SPIONs approved by the FDA in 1996 for clinical use has offered better imaging of the hepatobiliary system compared to gadolinium contrast agents [156]. However, like gadolinium, SPION imaging mainly relies on the different sources of blood supply between liver cancer tissues and normal liver tissues, lacking specificity. Therefore, the development of nanoprobes targeting specific HCC biomarkers aims to achieve early diagnosis of HCC, a direction many researchers are pursuing [157]. With the progression of research on the tumor microenvironment of HCC, an increasing number of molecular targets are being explored for molecular imaging. For instance, alpha-fetoprotein (AFP), GPC3, and FR found on the surface of tumor cells, but seldomly or not expressed in healthy cells, are targeted for imaging. Fe_3_O_4_ core/Au shell NP complexes (FANP) surface-modified with GPC3 binding peptides (GBP), observed through MR imaging, have shown specific accumulation in HepG2 tumors [109,158,159].

### 5.2 Nanotechnology combined with multimodal imaging for the diagnosis of HCC

With advancements in nanotechnology, a variety of nanoscale contrast agents have been developed for multimodal imaging. This approach synergistically combines the advantages of two or more imaging modalities, overcoming the limitations of single-mode imaging and offering more accurate diagnostic methods for HCC. For instance, lactose-modified polyethyleneimine encapsulated AuNPs have been utilized for targeted CT-MR dual-modal imaging of human HCC [160]. Similarly, the integration of NIR photoacoustic imaging and fluorescence imaging (FLI) offers high spatial and temporal resolution, outstanding optical contrast, and deep penetration, holding promise for accurate and sensitive diagnosis of HCC [161].

### 5.3 Application of nanomaterial technology in the diagnosis of serological and molecular targeted markers of liver cancer

Currently developed biomarkers for liver cancer include GPC-3, Osteopontin, Golgi Protein 73, and PIVKA, with alpha-fetoprotein (AFP) being the most commonly used. As the serum levels of these tumor markers are typically low, more sensitive detection methods are needed for early diagnosis of HCC. Nanomaterials can enhance the surface area of sensors, improving their transduction capability and overall sensitivity. For instance, metal NPs have been utilized for the electrochemical detection of GPC-3 [104].

## 6 The role of nanotechnology in the integrated diagnosis and treatment of liver cancer

**Figure 5:**
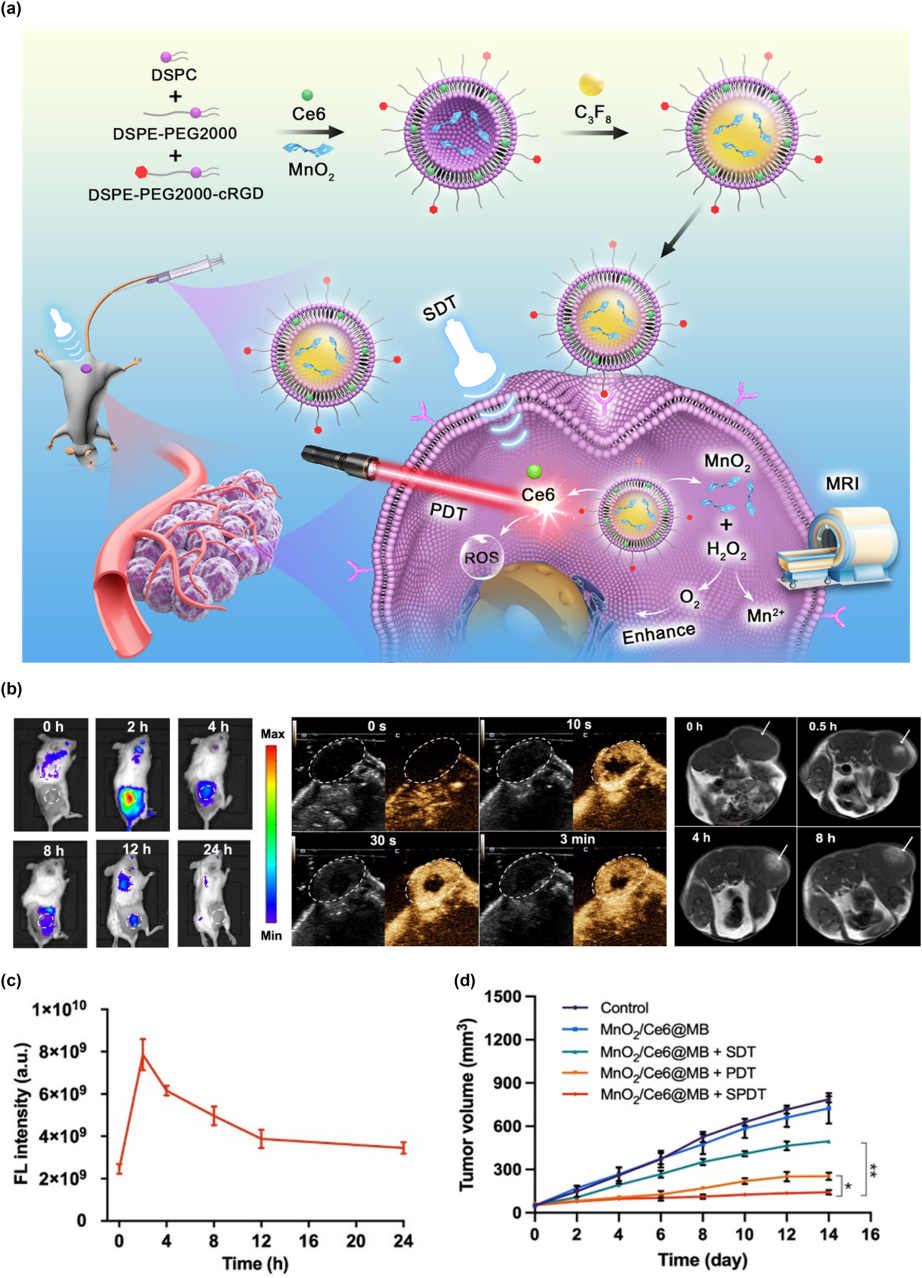
(a) Schematic illustration of the MnO_2_/Ce6@MB-mediated TNBC imaging and treatment. (b) Noninvasively targeted fluorescence imaging, US imaging, and MR imaging *in vivo*. (c) Semi-quantification of (b). (d) 4T1 tumor growth curve with time after the different treatments. [162] (Open Access). Copyright from Royal Society Of Chemistry.

By integrating imaging probes with therapeutic agents on a single nanoplatform, nanotechnology enables the convergence of diagnosis and treatment, as well as real-time monitoring of therapeutic outcomes. This offers a new, multifunctional platform for early diagnosis and precise treatment of liver cancer. Early diagnosis of liver cancer relies on imaging modalities such as MRI, CT, enhanced US, photoacoustic imaging (PAI), fluorescence imaging (FLI), and Raman imaging, with FLI, MRI, and enhanced US being the most commonly used. Treatment modalities include chemotherapy, gene therapy, immunotherapy, photodynamic therapy, SDT, PTT, and radiotherapy. Thanks to the progress in nanomedicine, many NPs have been modified into multifunctional nanoassemblies that can precisely deliver chemotherapy drugs, gene regulatory drugs to tumor tissues, and serve as photothermal agents, photosensitizers, and sonosensitizers in combination with PDT/PTT/SDT for treating liver cancer. These NPs can also be used in MRI, FLI, PAI, CT, and enhanced US imaging, facilitating an integrated approach to the diagnosis and treatment of liver cancer. Li et al. [162] developed a multifunctional therapeutic diagnostic NP platform (MnO_2_/Ce6@MBs) that can be used to enhance SPDT’s precise treatment of triple-negative breast cancer (TNBC). Meanwhile, the NP platform, consisting of three contrast agents, MN-2+, Ce6, and microvesicle, exhibits high-performance multimodal imaging in FL/MR/US imaging. It can guide the subsequent course of treatment (Figure 5).

## 7 Summary and outlook

In the treatment of liver cancer, NPs can be modified into multifunctional nanocomponents, which can be used as carriers of chemotherapy drugs, targeted drugs, gene therapy drugs, and immunotherapy drugs. They can also be combined with various treatments to reduce toxicity to normal liver cells, reduce drug resistance, and improve survival. These nanocomponents also have multiple diagnostic imaging modes for HCC, enabling comprehensive diagnostic and therapeutic functions. The advent of nanotechnology has marked significant progress in diagnosing and treating liver cancer, combining its unique properties with drugs for imaging and treating HCC, aiming at facilitating precise treatment [163]. Nano-based contrast agents not only reduce the drug concentration needed for MRI/CT/FLI-enhanced US imaging of HCC but also enhance resolution. NPs can sensitively detect AFP, GPC-3, and CTC in serological markers, aiding in the early diagnosis of HCC.

Nanomedicine research primarily focuses on the selective targeting of diseases and increasing the concentration of anticancer drugs at tumor sites. Yet, certain areas remain unaddressed, such as the evolving nature of anticancer drugs, the significant differences between various drugs, and the increased complexity in researching and developing nanocarrier systems due to their mechanisms of action and pharmacokinetics. Moreover, many nanomedicines lack standardized biosafety assessments, and their long-term toxicity cannot be ignored. And, so far, only a minority of NP-related products have entered clinical trials or the market. Despite the current shortcomings of nanomedical technologies in clinical applications, it is believed that with the concerted effort of numerous researchers, these challenges will be overcome in the near future, improving the quality and making nanomedicine an indispensable part of cancer diagnosis and treatment.

## Supplementary Material

Supplementary material
