# Supplementary material for: Research progress of nanoparticles in diagnosis and treatment of hepatocellular carcinoma
Source: Open Life Sci. 2024 Aug 23;19(1):20220932. doi: 10.1515/biol-2022-0932 (PMC11365471; doi:10.1515/biol-2022-0932)
Supplement: Supplementary material [file biol-2022-0932-sm.pdf]

# Supplementary material

**Table S1:** Therapeutic Nanocarriers delivery agents for hepatocellular carcinoma

| Nanoparticle | Drugs | Nanoplatform | Target cell | Anticancer mechanism | Ref. |
|---|---|---|---|---|---|
| Gold NPs | Sorafenib; doxorubicin | AuNPs-anti-miR221; DOX-loaded PNV; Au@16-Ph-16/DNA-Doxo | HepG2;Huh7; Bel-7402; | Delivery of drugs and gene therapy; PTT combine CT; | [7–9] |
| Silver NPs | — | Ag-polyvinylpyrrolidone | HepG2 | Oxidative stress | [10] |
| Silica NPs | — | M-MSNs | HepG2 | Mediated suicide gene therapy | [11] |
| Calcium NPs | Sorafenib | miR-375/Sf-LCC NPs | HepG2 | Codelivery of drugs and gene | [12] |
| Graphene Oxide-Based NPs | Sorafenib | GO-PEI-PEG | Hepatoma cells | Delivery of drugs; CT; Immunotherapy;ROS | [13] |
| SPIONs | Sorafenib | — | HepG2 | Apoptosis and autophagy | [14,15] |
| Metal oxide NPs (Iron, Zinc,Alumina) | Sorafenib;5-FU; Doxorubicin | SOR-PEG@Fe3O4 ; FPEGSO-MLDH ; FPVASOMLDH;PVA/ 5FU/Zn/Al-LDH; sMAG@PDA@DD5; NP | 3T3; HepG2 | Delivery of drugs; CT combine PTT | [16–18] |
| Liposomes | Lonidamine;sorafenib;VEGFsiRNA cisplatin;Curcumin FsiRNA | Lip-IR780/LND; CMCS-SiSf-CL; CDDP/CUR-Lip | LM3; HepG2; | PDT combine PTT; delivery of drugs and gene therapy;CT | [5,19,20] |
| Lipid NPs | doxorubicin; miR-375; bortezomib | LCC-DOX/miR-375 NPs; Gal-SLNs/BTZ | HepG2 | Delivery of drugs and gene therapy | [2,21] |
| Polymeric | Ursolic acid; Sorafenib | UA-PMs; sorafenib/PEG-PCL | HepG2 | Delivery of drugs and anti-HCC;CT | [22,23] |
| Chitosan NPs | Sorafenib; Triptolide | SF–CS NPs; GC-TP-NPs | SMMC-7721; HepG2 | Delivery of drugs and anti-HCC; blocking TNF/NF-κB/BCL2 | [4,24] |
| Bacterial magnetosomes | Doxorubicin;transferrin | Tf-BMs-DOX; PGCshRNA-GPC3 | Huh-7 | Delivery of drugs and gene therapy | [3,6] |
| Exosomes(clos | miR-26 a | — | HepG2 | Gene delivery and therapy | [25] |

Doxo: DNA and doxorubicin; DOX: Doxorubicin; SOR/SF/Sf: sorafenib; LND: Lonidamine; Lip: Liposomes; CMCS: carboxymethyl chitosan; SiSf-CL:Sf and Cy3-siRNA co-loaded cationic liposomes; CDDP: cisplatin; CUR: Curcumin; LCC: lipid-coated calcium carbonate; SLNs: solid lipid nanoparticles; Gal: galactose; BTZ: bortezomib; UA: Ursolic acid; PMs: Polymeric micelles; PEGPCL: PEG-poly (ε-caprolactone); CS: Chitosan; TP: Triptolide; GC: galactosylated chitosan; MLDH: magnesium-aluminum-layered double hydroxide; Tf: transferrin; BMs: bacterial magnetosomes; PEG: polyethylene glycol; DD5: dendrimers g.5.0; Mal: maleimide; sMAG: spheres magnetite; GO: graphene oxide; LCC：Lipid calcium carbonate; M-MSNs: magnetic mesoporous silica nanoparticles; PVA: polyvinyl alcohol; LDH: layered double hydroxide; PDA: polydopamine.

**Table S2:** Advantages, and disadvantages of different types of nanoparticles

| Nanoparticle type | Advantages | Disadvantages |
|---|---|---|
| Carbon-based NPs | high surface area; excellent conductivity; effective catalytic properties and biocompatibility [28,29] | Bio-corona formation; Toxicity; Environmental toxicity [30] |
| Liposomes | Hydrophillic and hydrophobic; drug encapsulation; Biocompatibility; Low imunogenicity; Biodegradable; nontoxic [31] | Low encapsulation efficiency; Short shelf-life; Accelerated blood clearance [26] |
| Iron oxide NPs | has magnetic; biomedical properties; enhances biocompatibility; bioavailability and bioactivity [40] | cytotoxicity; Internal iron disorder; protein corona [40] |
| SPIONs | External guidance<br>Hyperthermia applications<br>High stability | Potential toxicity; Laborious synthesis; liable to form agglomerates; get easily oxidized in the; Easily metabolized; swallowed by macrophages air [33] |
| Polymeric micelles | Free from gastrointestinal system; controlled drug release; decreased side effects; high bioavailability; Drug loading [34] | Low drug loading; Low stability [27,36] |
| AuNPs | Facile synthesis; surface functionalization; superior optical; electronic properties [35,36] | Potential genotoxicity; High costs of raw materials [1] |
| graphene NPs | Good mechanical strength; aspect ratio; conductivity; chemical stability; biocompatibility; high surface area [37] | Low solubility; non-homogeneity in size; metal impurities; colloidal stability; poor chemical stability; Sensitive to oxidation environment [37] |
| Silica | Modifiable surface with various ligands; adjustable particle size; large surface area; tunable pore structure; high drug-loading capacity [38,39] | Potential toxicity; Slow biodegradability [38] |
| Silver NPs | Biocompatibility; Chemical stability; catalysis; good electrical conductivity; most notably antiviral; antibacterial; antifungal activity [39] | genetic toxicity; Easily affected by temperature particle size; Cytotoxicity; Cause inflammatory response [40,41] |

# References


[1] Graur F, Puia A, Mois EI, Moldovan S, Pusta A, Cristea C, et al. Nanotechnology in the diagnostic and therapy of hepatocellular carcinoma. Materials (Basel). 2022;15(11).

[2] Mostafaei F, Hemmati S, Valizadeh H, Mahmoudian M, Sarfraz M, Abdi M, et al. Enhanced intracellular accumulation and cytotoxicity of bortezomib against liver cancer cells using Nstearyl lactobionamide surface modified solid lipid nanoparticles. Int J Pharm. 2024;649:123635.

[3] Wang J, Geng Y, Zhang Y, Wang X, Liu J, Basit A, et al. Bacterial magnetosomes loaded with doxorubicin and transferrin improve targeted therapy of hepatocellular carcinoma. Nanotheranostics. 2019;3(3):284–98.

[4] Albalawi F, Hussein MZ, Fakurazi S, Masarudin MJ. Fabrication and characterization of nanodelivery platform based on chitosan to improve the anticancer outcome of sorafenib in hepatocellular carcinoma. Sci Rep. 2023;13(1):12180.

[5] Cheng Y, Zhao P, Wu S, Yang T, Chen Y, Zhang X, et al. Cisplatin and curcumin co-loaded nano-liposomes for the treatment of hepatocellular carcinoma. Int J Pharm. 2018;545(12):261–73.

[6] Lei CJ, Yao C, Pan QY, Long HC, Li L, Zheng SP, et al. Lentivirus vectors construction of SiRNA targeting interference GPC3 gene and its biological effects on liver cancer cell lines Huh-7. Asian Pac J Trop Med. 2014;7(10):780–6.

[7] Cai H, Yang Y, Peng F, Liu Y, Fu X, Ji B. Gold nanoparticles-loaded anti-miR221 enhances antitumor effect of sorafenib in hepatocellular carcinoma cells. Int J Med Sci. 2019;16(12):1541–8.

[8] Wang L, Yuan Y, Lin S, Huang J, Dai J, Jiang Q, et al. Photothermo-chemotherapy of cancer employing drug leakage-free gold nanoshells. Biomaterials. 2016;78:40–9.

[9] Giráldez-Pérez RM, Grueso E, Montero-Hidalgo AJ, Luque RM, Carnerero JM, Kuliszewska E, Prado-Gotor R. Gold Nanosystems covered with doxorubicin/DNA complexes: a therapeutic target for prostate and liver cancer. Int J Mol Sci. 2022;23(24).

[10] Kitchin KT, Richards JA, Robinette BL, Wallace KA, Coates NH, Castellon BT, et al. Biochemical effects of silver nanomaterials in human hepatocellular carcinoma (HepG2) cells. J Nanosci Nanotechnol. 2020;20(9):5833–58.

[11] Wang Z, Chang Z, Lu M, Shao D, Yue J, Yang D, et al. Shape-controlled magnetic mesoporous silica nanoparticles for magnetically-

mediated suicide gene therapy of hepatocellular carcinoma. Biomaterials. 2018;154:147–57.
[12] Zhao P, Li M, Wang Y, Chen Y, He C, Zhang X, et al. Enhancing antitumor efficiency in hepatocellular carcinoma through the autophagy inhibition by miR-375/sorafenib in lipidcoated calcium carbonate nanoparticles. Acta Biomater. 2018;72:248–55.
[13] Li Z, Bu J, Zhu X, Zhou H, Ren K, Chu PK, et al. Anti-tumor immunity and ferroptosis of hepatocellular carcinoma are enhanced by combined therapy of sorafenib and delivering modified GO-based PD-L1 siRNAs. Biomater Adv. 2022;136:212761.
[14] Tom G, Philip S, Isaac R, Praseetha PK, Jiji SG, Asha VV. Preparation of an efficient and safe polymeric-magnetic nanoparticle delivery system for sorafenib in hepatocellular carcinoma. Life Sci. 2018;206:10–21.
[15] Ebadi M, Rifqi Md Zain A, Tengku Abdul Aziz TH, Mohammadi H, Tee CAT, Rahimi Yusop M. Formulation and characterization of Fe (3)O(4)@PEG nanoparticles loaded sorafenib; molecular studies and evaluation of cytotoxicity in liver cancer cell lines. Polymer (Basel). 2023;15(4).
[16] Ebadi M, Bullo S, Buskara K, Hussein MZ, Fakurazi S, Pastorin G. Release of a liver anticancer drug, sorafenib from its PVA/LDH- and PEG/LDH-coated iron oxide nanoparticles for drug delivery applications. Sci Rep. 2020;10(1):21521.
[17] Ebadi M, Bullo S, Buskaran K, Hussein MZ, Fakurazi S, Pastorin G. Dual-functional iron oxide nanoparticles coated with polyvinyl alcohol/5-fluorouracil/zinc-aluminium-layered double hydroxide for a simultaneous drug and target delivery system. Polymer (Basel). 2021;13(6).
[18] Jędrzak A, Grześ Kowiak BF, Golba K, Coy E, Synoradzki K, Jurga S, et al. Magnetite nanoparticles and spheres for chemo- and photothermal therapy of hepatocellular carcinoma in vitro. Int J Nanomed. 2020;15:7923–36.
[19] Lei L, Dai W, Man J, Hu H, Jin Q, Zhang B, Tang Z. Lonidamine liposomes to enhance photodynamic and photothermal therapy of hepatocellular carcinoma by inhibiting glycolysis. J Nanobiotechnol. 2023;21(1):482.
[20] Yao Y, Wang T, Liu Y, Zhang N. Co-delivery of sorafenib and VEGF-siRNA via pHsensitive liposomes for the synergistic treatment of hepatocellular carcinoma. Artif Cell Nanomed Biotechnol. 2019;47(1):1374–83.
[21] Zhao P, Wu S, Cheng Y, You J, Chen Y, Li M, et al. MiR-375 delivered by lipid-coated doxorubicin-calcium carbonate nanoparticles overcomes chemoresistance in hepatocellular carcinoma. Nanomedicine. 2017;13(8):2507–16.
[22] Zhou M, Yi Y, Liu L, Lin Y, Li J, Ruan J, Zhong Z. Polymeric micelles loading with ursolic acid enhancing anti-tumor effect on hepatocellular carcinoma. J Cancer. 2019;10(23):5820–31.
[23] Su Y, Wang K, Li Y, Song W, Xin Y, Zhao W, et al. Sorafenib-loaded polymeric micelles as passive targeting therapeutic agents for hepatocellular carcinoma therapy. Nanomedicine (Lond). 2018;13(9):1009–23.
[24] Zhang YQ, Shen Y, Liao MM, Mao X, Mi GJ, You C, et al. Galactosylated chitosan triptolide nanoparticles for overcoming hepatocellular carcinoma: Enhanced therapeutic efficacy, low toxicity, and validated network regulatory mechanisms. Nanomedicine. 2019;15(1):86–97.
[25] Liang G, Kan S, Zhu Y, Feng S, Feng W, Gao S. Engineered exosome-mediated delivery of functionally active miR-26a and its enhanced suppression effect in HepG2 cells. Int J Nanomed. 2018;13:585–99.
[26] Gavas S, Quazi S, Karpiński TM. Nanoparticles for cancer therapy: current progress and challenges. Nanoscale Res Lett. 2021;16(1):173.
[27] Majumder N, Das NG, Das SK. Polymeric micelles for anticancer drug delivery. Ther Delivery. 2020;11(10):613–35.
[28] Fritea L, Banica F, Costea TO, Moldovan L, Dobjanschi L, Muresan M, Cavalu S. Metal nanoparticles and carbon-based nanomaterials for improved performances of electrochemical (bio) sensors with biomedical applications. Mater (Basel). 2021;14(21.
[29] Kaurav H, Verma D, Bansal A, Kapoor DN, Sheth S. Progress in drug delivery and diagnostic applications of carbon dots: a systematic review. Front Chem. 2023;11:1227843.
[30] Debnath SK, Srivastava R. Drug delivery with carbon-based nanomaterials as versatile nanocarriers: progress and prospects. 2021
[31] Dymek M, Sikora E. Liposomes as biocompatible and smart delivery systems - the current state. Adv Colloid Interface Sci. 2022;309:102757.
[32] Fernández-Bertólez N, Costa C, Brandão F, Teixeira JP, Pásaro E, Valdiglesias V, Laffon B. Toxicological aspects of iron oxide nanoparticles. Adv Exp Med Biol. 2022;1357:303–50.
[33] Khan SA, Sharma R. Super para-magnetic iron oxide nanoparticles (SPIONs) in the treatment of cancer: challenges, approaches, and its pivotal role in pancreatic, colon, and prostate cancer. Curr Drug Delivery. 2023;20(6):643–55.
[34] Jain A, Bhardwaj K, Bansal M. Polymeric micelles as drug delivery system: recent advances, approaches, applications and patents. Curr Drug Saf. 2024;19(2):163–71.
[35] Tabatabaei MS, Islam R, Ahmed M. Applications of gold nanoparticles in ELISA, PCR, and immuno-PCR assays: A review. Anal Chim Acta. 2021;1143:250–66.
[36] Nooranian S, Mohammadinejad A, Mohajeri T, Aleyaghoob G, Kazemi Oskuee R. Biosensors based on aptamer-conjugated gold nanoparticles: A review. Biotechnol Appl Biochem. 2022;69(4):1517–34.
[37] Mahor A, Singh PP, Bharadwaj P, Sharma N, Yadav S, Rosenholm JM, et al. Carbon-based nanomaterials for delivery of biologicals and therapeutics: a cutting-edge technology. C – J Carbon Res. 2021;7(1):19.
[38] Zhang Y, Lin X, Chen X, Fang W, Yu K, Gu W, et al. Strategies to regulate the degradation and clearance of mesoporous silica nanoparticles: a review. Int J Nanomed. 2024;19:5859–78.
[39] Gong X, Jadhav ND, Lonikar VV, Kulkarni AN, Zhang H, Sankapal BR, et al. An overview of green synthesized silver nanoparticles towards bioactive antibacterial, antimicrobial and antifungal applications. Adv Colloid Interface Sci. 2024;323:103053.
[40] Egbuna C, Parmar VK, Jeevanandam J, Ezzat SM, Patrick-Iwuanyanwu KC, Adetunji CO, et al. Toxicity of nanoparticles in biomedical application: nanotoxicology. J Toxicol. 2021;2021:9954443.
[41] Barkat MA, Beg S, Naim M, Pottoo FH, Singh SP, Ahmad FJ. Current progress in synthesis, characterization and applications of silver nanoparticles: precepts and prospects. Recent Pat Anti-infect Drug Discovery. 2018;13(1):53–69.
